# Synthesis and Biological Properties of Pyranocoumarin Derivatives as Potent Anti-Inflammatory Agents

**DOI:** 10.3390/ijms241210026

**Published:** 2023-06-12

**Authors:** Su Ji Min, Heesu Lee, Myoung-Sook Shin, Jae Wook Lee

**Affiliations:** 1 College of Korean Medicine, Gachon University, Seongnam-si 13120, Republic of Korea; 2 Department of Anatomy, College of Dentistry, Gangneung Wonju National University (GWNU), Gangneung-si 25457, Republic of Korea; nightsu@gwnu.ac.kr; 3 Natural Product Research Center, Korea Institute of Science and Technology (KIST), Gangneung-si 25451, Republic of Korea; 4 Division of Bio-Medical Science and Technology, KIST School, University of Science and Technology (UST), Gangneung-si 25451, Republic of Korea

**Keywords:** Coumarins, RAW264.7 macrophages, lipopolysaccharide

## Abstract

This study aimed to synthesize 23 coumarin derivatives and analyze their anti-inflammatory effects on lipopolysaccharide (LPS)-induced inflammation in RAW264.7 macrophages. A cytotoxicity test performed on LPS-induced RAW264.7 macrophages revealed that none of the 23 coumarin derivatives were cytotoxic. Among the 23 coumarin derivatives, coumarin derivative 2 showed the highest anti-inflammatory activity by significantly reducing nitric oxide production in a concentration-dependent manner. Coumarin derivative 2 inhibited the production of proinflammatory cytokines, including tumor necrosis factor alpha and interleukin-6, and decreased the expression level of each mRNA. In addition, it inhibited the phosphorylation of extracellular signal-regulated kinase, p38, c-Jun NH2-terminal kinase, nuclear factor kappa-B p65 (NF-κB p65), and inducible nitric oxide synthase. These results indicated that coumarin derivative 2 inhibited LPS-induced mitogen-activated protein kinase and NF-κB p65 signal transduction pathways in RAW264.7 cells, as well as proinflammatory cytokines and enzymes related to inflammatory responses, to exert anti-inflammatory effects. Coumarin derivative 2 showed potential for further development as an anti-inflammatory drug for the treatment of acute and chronic inflammatory diseases.

## 1. Introduction

Coumarins and pyrans are heterocyclic molecules that exhibit several biological activities and are usually found in natural plants, synthetic drugs, and drug candidates with important biological activities [1,2]. Pyranocoumarins may also be significant as therapeutic agents [3,4], including anticancer [5], antiviral [6], antimicrobial [7], anti-inflammatory effects [8], anticoagulants, and potent cyclooxygenase-2 inhibitors [9].

**Figure 1:**
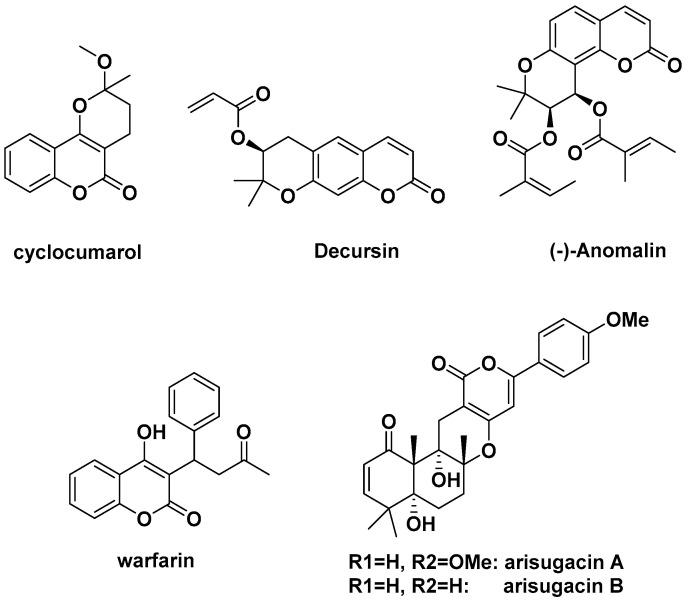
Structures of biologically active pyranocoumarins.

Decursin and its derivatives from the herb *Angelica gigas* possess potent antiandrogen receptor activities and induce cell cycle arrest and caspase-mediated apoptosis [10]. Anomalin, isolated from *Angelica anomala* Ave-Lal for the first time, has been shown to have many biological activities, including anti-inflammatory, analgesic, antioxidant, antitumor, hepatoprotective, and neuroprotective activities [11]. Arisugacin A and B, isolated from a culture broth of *Penicillium* sp. FO-4259, have been shown to have selective inhibitory effects against acetylcholinesterase with half maximal inhibitory concentration (IC_50_) values of 1 and 26 nM, respectively [12,13,14]. Therefore, we expect that the newly synthesized pyranocoumarins may also show interesting biological activities (Figure 1).

**Scheme 1:**
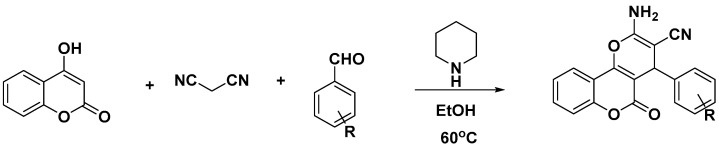
Synthesis of pyranocoumarin derivatives.

**Figure 2:**
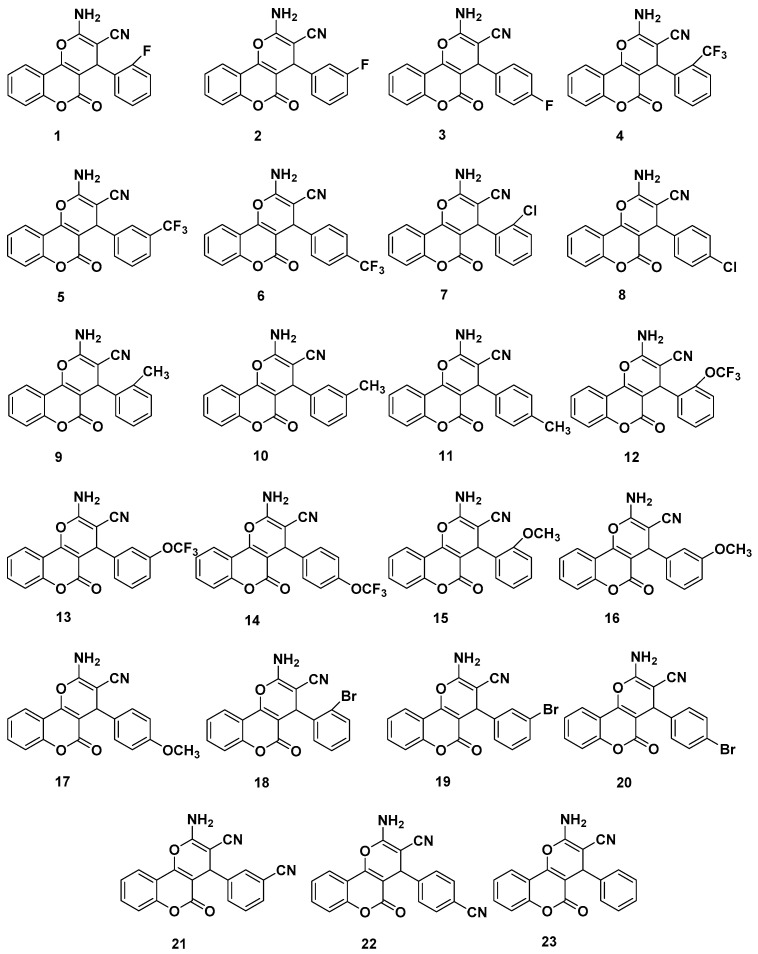
Structures of pyranocoumarins.

Pyranocoumarins can be synthesized via the three-component Diels–Alder reaction among 4-hydroxycoumarin, malononitrile, and various benzaldehydes (Scheme 1). To expand the diversity of pyranocoumarins, various benzaldehydes were used for their synthesis (Figure 2). The detailed synthetic procedure for pyranocoumarin derivatives is described in the Supporting Information (Supplementary S1). The synthesized pyranocoumarins were identified via nuclear magnetic resonance and liquid chromatography–mass spectrometry (LC–MS).

Inflammation is an immune response to protect the body against trauma and tissue damage caused by physical and chemical stimuli and is mediated by various immune cells [15,16,17]. The inflammatory response helps in the prevention of diseases [18,19]. During inflammation, macrophages recognize lipopolysaccharide (LPS) through Toll-like receptors expressed on the cell surface [20,21]. LPS is present in the outer membrane of Gram-negative bacteria and activates nuclear factor kappa-B (NF-κB) to activate inducible nitric oxide synthase (iNOS) and COX-2 [22,23]. In addition, when LPS binds to TLR4, which is expressed on the surface of macrophages, nitric oxide (NO) and prostaglandin E2, which are inflammatory mediators, as well as tumor necrosis factor-α (TNF-α), interleukin-6 (IL-6), and interleukin-1β (IL-1β) induce inflammation [24,25,26]. Mitogen-activated protein kinases (MAPKs), such as extracellular signal-regulated kinase (ERK), c-Jun NH2-terminal kinase (JNK), and p38 kinases (p38), are proteins involved in intracellular signal transduction that are activated during inflammatory responses and by stimuli, such as LPS [27,28]. In addition, p65, a subunit of NF-κB, is a transcription factor involved in various gene expressions, such as immune or inflammatory responses. Under normal conditions, p65 is bound to the inhibitory kappa-B (Iκ-B) protein in the cellular cytoplasm. When cells are stimulated by NF-κB and LPS, Iκ-Bα is phosphorylated and separated from the NF-κB subunit p65, and p65 moves into the nucleus to induce the gene expression of various inflammatory mediators, such as iNOS and cytokines, and increase inflammatory substances [29,30,31]. NO is a highly reactive radical. When macrophages are activated, NO is produced from iNOS, and at low concentrations, it has important physiological roles, such as signal transduction and immune function. However, excessive expression of iNOS reportedly causes malignant tumors (brain, lung, breast, pancreatic, and prostate cancer), gene mutations, and tissue and nerve damage [24,25]. Considering that iNOS is responsible for the excessive production of NO, which causes these inflammatory diseases, substances that inhibit iNOS gene expression are highly likely to serve as inflammatory control agents [32,33]. In addition, overproduction of inflammatory mediators and cytokines reportedly causes arthritis, multiple sclerosis, asthma, inflammatory bowel disease, and atherosclerosis [34,35]. Recently, with an increase in various inflammatory diseases, interest in bioactive substances capable of inhibiting the production of inflammatory mediators and cytokines, such as iNOS, has increased.

In the present study, 23 types of coumarin derivatives were synthesized, and their NO inhibitory activity was analyzed to determine their anti-inflammatory activity. Among them, coumarin derivative 2, which showed the highest nitric oxide inhibitory activity, was used to elucidate the intracellular mechanism of anti-inflammatory activity in RAW264.7 macrophages.

## 2. Results

### 2.1. Effects of Coumarin Derivatives on the Viability of RAW264.7 Cells

To investigate the anti-inflammatory activity of coumarin derivatives, cell viability was analyzed by treating RAW264.7 cells with 23 types of coumarin derivatives at concentrations of 20, 40, and 80 μM for 2 h and with LPS for 20 h. As shown in Table 1, no cytotoxicity was observed when the cells were treated with coumarin derivatives (Table 1). Based on these results, additional experiments were performed at concentrations of 20–80 μM, as these concentrations were not cytotoxic.

### 2.2. Effects of Coumarin Derivatives on Nitric Oxide Production in LPS-Stimulated RAW264.7 Cells

NO, synthesized from L-arginine by the enzymatic activity of iNOS, exhibits various functions and roles in the immune system [36]. A high concentration of NO promotes the synthesis of inflammatory mediators, leading to an inflammatory response, and has been reported to be a cause of various immune diseases [37]. Therefore, this study was conducted to investigate the effect of coumarin derivatives on the production of NO induced by LPS stimulation in RAW264.7 cells. The cells were treated with coumarin derivatives at concentrations of 20, 40, and 80 μM for 2 h. Then, the cells were treated with LPS. After 20 h, the culture supernatant was recovered, and the amount of NO was measured. Compared with LPS-only treatment, coumarin derivatives 2, 10, and 19 inhibited NO secretion in a concentration-dependent manner (Table 2). Among these coumarin derivatives, meta-substituted derivatives 2 (–F), 10 (–CH_3_), and 19 (–Br) showed dose-dependent anti-inflammatory effects. The anti-inflammatory activity of these derivatives depended on the position of the substituted group rather than the electron density of the substituted group. Compared with meta-substituted derivatives, derivatives 5 (–CF_3_) and 13 (–OCF_3_) showed almost no anti-inflammatory effects. Derivative 21 (–CN) showed weak anti-inflammatory effects. Ortho-substituted derivatives 1 (–F), 9 (–CH_3_), and 18 (–Br) showed almost no anti-inflammatory effect. However, derivatives 12 (–CF_3_) and 15 (–OCH_3_) showed anti-inflammatory effects. Among para-substituted derivatives, derivatives 6 (–CF_3_), 14 (–OCF_3_), 17 (–OCH_3_), and 22 (–CN) showed weak anti-inflammatory effects at high concentrations.

Among them, coumarin derivative 2 showed the greatest inhibitory effect by significantly reducing NO production in a concentration-dependent manner. Therefore, since coumarin derivative 2 inhibited NO production (IC_50_: 33.37 μM), it was considered to be an effective anti-inflammatory agent, and additional experiments were performed using coumarin derivative 2 to elucidate the molecular mechanism underlying the induction of the expression of various inflammatory factors.

### 2.3. Analysis of the Effect of Coumarin Derivative 2 on IL-6 and TNF-α Production and mRNA Expression in RAW264.7 Cells

Macrophages stimulated by pathogens and cytokines are activated and play an important role in regulating the immune response, inflammatory response, and metabolism [31,33,35]. LPS-activated RAW264.7 cells were treated with 20, 40, or 80 μM of coumarin derivative 2 and cultured for 24 h, and the levels of TNF-α and IL-6 in the culture medium were analyzed.

**Figure 3:**
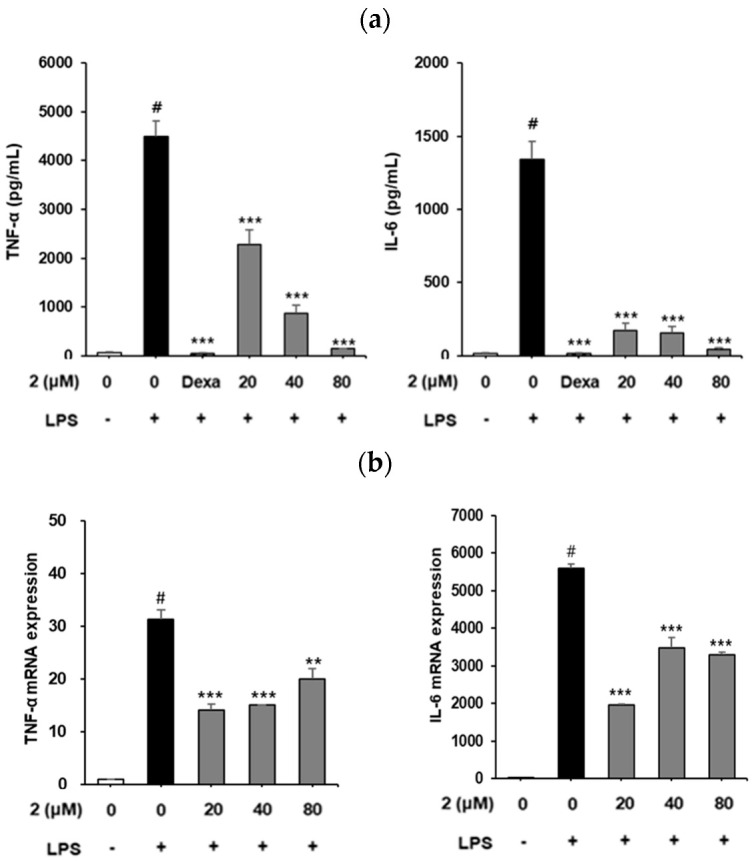
Effects of coumarin derivative 2 on IL-6 and TNF-α production and mRNA expression in LPS-stimulated RAW264.7 cells. (**a**) Effect of coumarin derivative 2 on TNF-α and IL-6 production in RAW264.7 cells. The RAW264.7 cells (1 × 10^5^/well, 96-well plates) were pretreated with coumarin derivative 2 (20, 40, and 80 μM) for 2 h and incubated with LPS (500 ng/mL) for 20 h. An ELISA kit was used to analyze cell supernatants for TNF-α and IL-6. (**b**) Effect of coumarin derivative 2 on mRNA expression of IL-6 and TNF-α in RAW264.7 cells. The RAW264.7 cells (2.0 × 10^6^ cells/6 cm dish) were pretreated with coumarin derivative 2 (20, 40, and 80 μM) for 2 h and incubated with LPS (500 ng/mL) for 6 h. IL-6 and TNF-α mRNA expression levels were measured via a real-time quantitative reverse transcription polymerase chain reaction. (−): nontreated group; (+): LPS-treated group; dexamethasone (Dexa, 100 μM): positive control. # *p* < 0.0001 vs. control group. *** *p* < 0.0001, ** *p* < 0.001 vs. LPS group.

As shown in Figure 3a, compared with the LPS treatment, the sample treatment group significantly reduced the LPS-induced IL-6 and TNF-α levels in a concentration-dependent manner at all concentrations (20, 40, and 80 μM). In addition, mRNA expression involved in IL-6 and TNF-α production was analyzed. The effect of LPS on mRNA expression was significantly suppressed after treatment with coumarin derivative 2 (Figure 3b).

These results indicate that coumarin derivative 2 possesses anti-inflammatory effects related to IL-6 and TNF-α production in RAW264.7 cells.

### 2.4. Effect of Coumarin Derivative 2 on iNOS Expression in LPS-Induced RAW264.7 Cells

**Figure 4:**
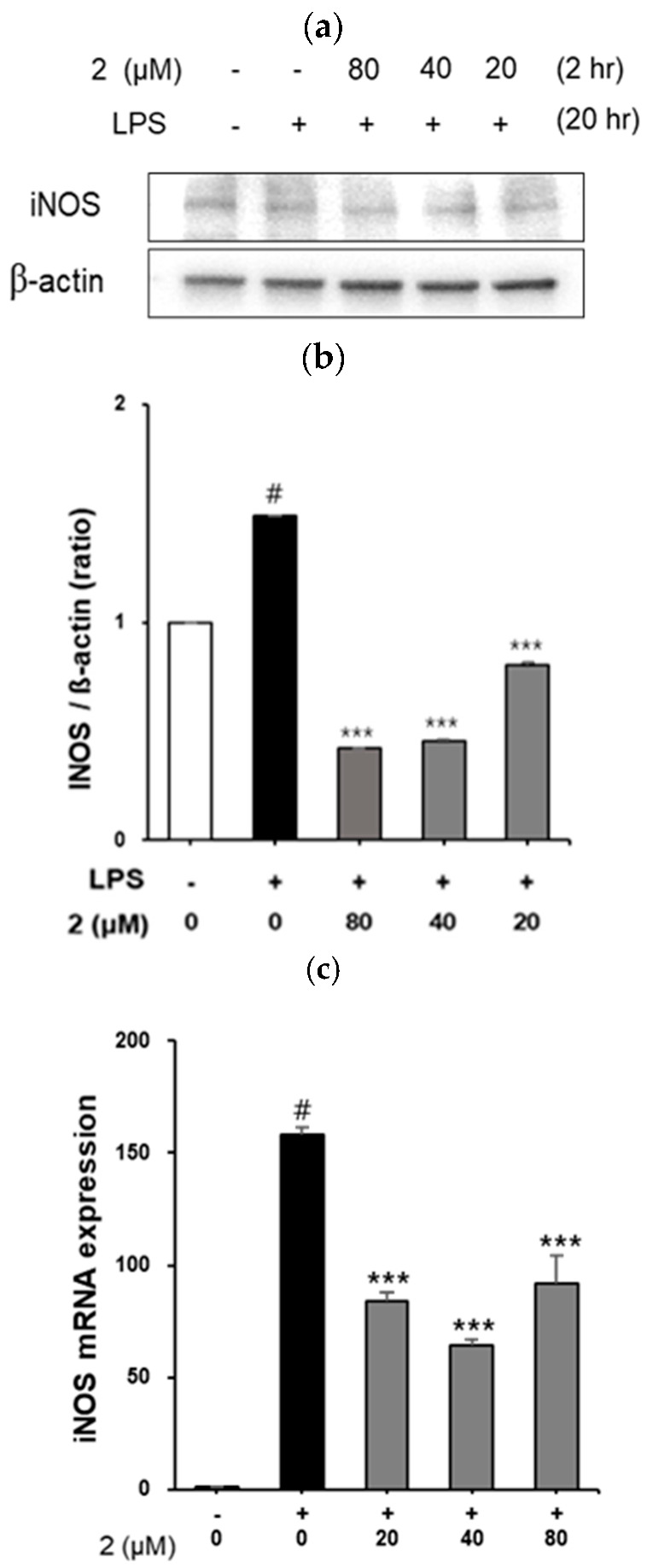
Effect of coumarin derivative 2 on iNOS protein expression in LPS-stimulated RAW264.7 cells. (**a**) RAW264.7 cells (2.0 × 10^6^ cells/6 cm dish) were pretreated with coumarin derivative 2 (20, 40, and 80 μM) for 2 h and incubated with LPS (500 ng/mL) for 20 h. Whole-cell lysates were then immunoblotted with the iNOS specific antibody. Actin was used for loading control. (**b**) iNOS protein expression levels and total protein were verified with Image J software 1.8.0. β-Actin was used as a control. (**c**) RAW264.7 cells (2.0 × 10^6^ cells/6 cm dish) were pretreated with coumarin derivative 2 (20, 40, and 80 μM) for 2 h and incubated with LPS (500 ng/mL) for 6 h. iNOS mRNA expression levels were measured via a real-time quantitative reverse transcription polymerase chain reaction. Data are presented as the mean ± standard deviation of three independent experiments. (−): nontreated group; (+): LPS-treated group. # *p* < 0.0001 vs. the control group. *** *p* < 0.0001 vs. the LPS group.

The inflammation-inducing gene iNOS is a known causative agent that induces an inflammatory response by inducing the activity of immune-related cells, such as macrophages, in a pathological environment, such as inflammation [34]. Reportedly, most NO produced in the inflammatory response is synthesized from L-arginine and O_2_ by the action of iNOS [36]. This means that iNOS expression is closely related to NO production. To investigate the effects of coumarin derivative 2 and their correlations, iNOS expression was confirmed after inducing inflammation for 24 h after LPS treatment. It was confirmed that phosphorylation was inhibited in the group pretreated with coumarin derivative 2 (Figure 4a). In addition, the mRNA expression involved in the production of iNOS was analyzed. The effect of LPS on mRNA expression was significantly suppressed after treatment with coumarin derivative 2 (Figure 4c), suggesting that the derivative has anti-inflammatory effects related to iNOS production in RAW264.7 cells. The NO inhibitory activity of coumarin derivative 2 is due to the decrease in mRNA expression of iNOS, and it is predicted that the compound can serve as an effective anti-inflammatory agent by targeting the underlying inflammatory mechanism of action.

### 2.5. Analysis of the Effect of Coumarin Derivative 2 on MAPKs and NF-kB p65 Phosphorylation in LPS-Induced RAW264.7 Cells

**Figure 5:**
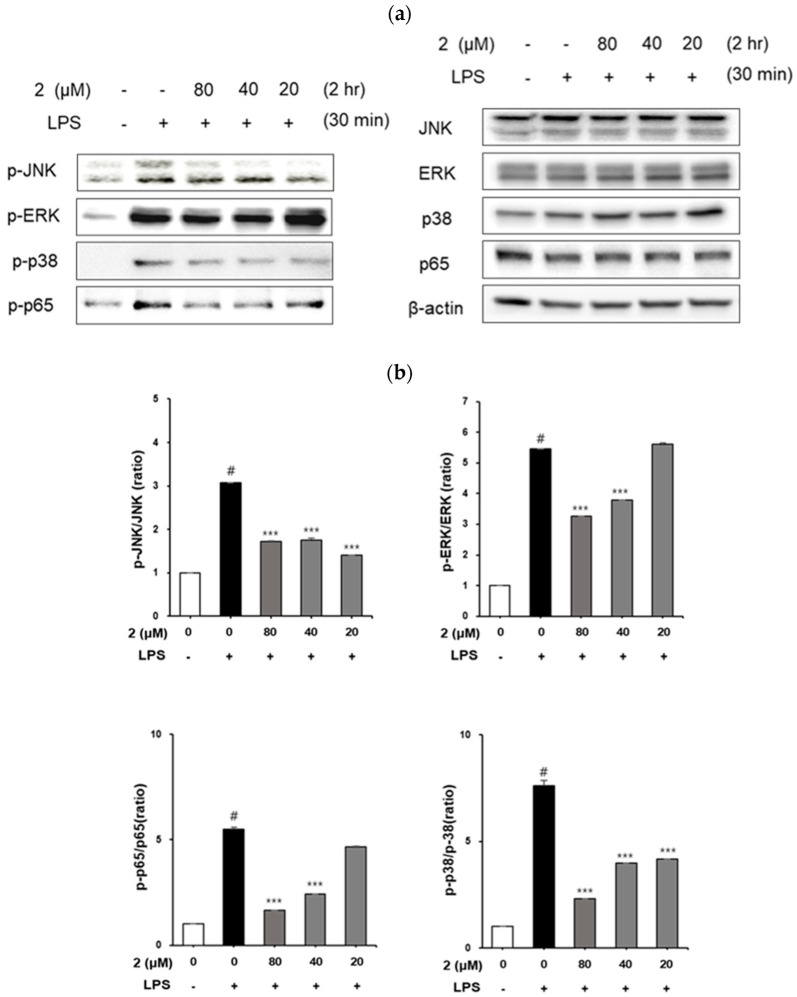
Inhibitory effects of coumarin derivative 2 on LPS-induced phosphorylation of MAPKs and NF-κB p65 in RAW264.7 cells. (**a**) RAW264.7 cells (2.0 × 10^6^ cells/6 cm dish) were pretreated with coumarin derivative 2 (20, 40, and 80 μM) for 2 h and incubated with LPS (500 ng/mL) for 30 min. Whole-cell lysates were then immunoblotted with the specific antibodies indicated in the left panel. (**b**) phospho-JNK, phospho-ERK, phospho-p38, and phospho-p65 protein expression levels and total protein were verified by Image J software. Actin was used as the control. Data are presented as the mean ± standard deviation of three independent experiments. (−): nontreated group; (+): LPS-treated group. # *p* < 0.0001 vs. the control group. *** *p* < 0.0001 vs. the LPS group.

LPS-induced RAW264.7 cells secrete cytokines through various mechanisms, and MAPKs and NF-κB are typical LPS-related signaling pathways. The phosphorylation of ERK, JNK, and p38 was strongly induced by LPS, whereas it was inhibited by coumarin derivative 2 (Figure 5a). Next, the NF-κB pathway, an inflammatory pathway activated by LPS, was analyzed. When the cells are normal, IκBα protein is present in the cytoplasm in a complex with p65 and p50, but it becomes phosphorylated when stimulated by LPS [28,30]. Simultaneously, phosphorylation of p65 occurs, and the phosphorylated protein is transported to the nucleus, where it acts as a transcription factor. When treated with coumarin derivative 2, p65 phosphorylation was inhibited (Figure 5a).

Based on these results, coumarin derivative 2 was predicted to suppress the production of inflammatory factors, such as NO, IL-6, and TNF-α, by inhibiting the phosphorylation of the JNK, ERK, p38, and NF-κB p65 pathways.

## 3. Discussion

Inflammatory response is a mechanism by which immune cells recognize the inflow of harmful external substances, such as viruses, into the body and secrete various inflammatory mediators and proinflammatory cytokines to protect the living body [25,38]. However, an excessive and continuous inflammatory response damages the mucous membrane and causes adverse effects, such as pain, swelling, and fever, resulting in functional disorders, various diseases, tissue damage, and genetic mutations [25]. Most of the currently used anti-inflammatory drugs cause side effects, such as decreased renal function, increased blood pressure, and gastrointestinal ulcers, as well as circulatory diseases, such as myocardial infarction, when consumed for a longer duration [39]. Thus, several studies have been conducted recently to develop new substances with low toxicity and excellent anti-inflammatory action using natural derivatives [40,41]. In the present study, we synthesized 23 types of coumarin derivatives and investigated their anti-inflammatory effects. The 23 types of coumarin derivatives were tested at various concentrations (20, 40, and 80 μM), and coumarin derivative 2, which had the highest anti-inflammatory activity without cytotoxicity, was considered an effective anti-inflammatory agent. The anti-inflammatory activities and mechanisms of coumarin derivative 2 were investigated in LPS-induced macrophages. LPS is present in the outer membrane of the cell walls of Gram-negative bacteria and is recognized by TLR4 on the surface of macrophages, leading to the subsequent initiation of an inflammatory signaling pathway [42]. Macrophages exposed to LPS produce various proinflammatory mediators, such as NO and iNOS, and proinflammatory cytokines, such as IL-6 and TNF-α [24,25]. NO is produced by NOS, and among NOS, iNOS produces a large amount of NO when induced by external stimuli or cytokines [43]. Excessive formation of highly reactive NO accelerates tissue damage and cytokine synthesis, intensifying inflammation [44]. iNOS expression and NO production are characteristic inflammatory responses of immune cells. Therefore, iNOS gene expression is an important mechanism by which inflammatory mediators are overproduced by NO and should be appropriately regulated to prevent chronic inflammation.

Treatment of LPS-induced macrophages with various concentrations (20, 40, and 80 μM) of coumarin derivative 2 showed that NO production was inhibited in a concentration-dependent manner (Table 2). In addition, the production of inflammatory mediators, such as NO, and inflammatory cytokines, such as TNF-a and IL-6, was significantly inhibited in a concentration-dependent manner (Figure 3a). Moreover, coumarin derivative 2 controlled excessive immune responses by suppressing the amount of mRNA expression (Figure 3b). Overall, coumarin derivative 2 was effective in reducing NO production. Therefore, inhibiting the production of inflammatory mediators and inhibiting the secretion of proinflammatory cytokines can reduce the inflammatory response, which is an essential indicator of anti-inflammatory properties, demonstrating the anti-inflammatory effect of coumarin derivative 2.

TLR4 increases MAPK phosphorylation by inflammatory substances (TNF-α, IL-6, and iNOS) to increase macrophage activity [45]. In addition, iNOS expressed by LPS regulates the expression of JNK, ERK, and p38 [46]. Coumarin derivative 2 significantly reduced the phosphorylation of JNK, ERK, and p38 in LPS-induced macrophages (Figure 5). These results imply that coumarin derivative 2 exhibits anti-inflammatory activity by blocking the MAPK signaling pathway. NF-κB plays an important role in inflammatory diseases and is a central transcription factor involved in the secretion of inflammatory mediators and cytokines [47]. In addition, NF-κB is present in the cytosol, and once cells are activated by LPS stimulation, IκB kinase rapidly phosphorylates and degrades IκB in the IκB/NF-κB complex. Consequently, NF-κB translocates to the nucleus and expresses inflammation-related genes [48]. When IκB is phosphorylated in the cytoplasm by LPS stimulation, p65 bound to IκB moves into the nucleus and acts as a transcription factor, inducing the expression of inflammatory cytokines and inflammatory factors; thus, inhibition of p65 phosphorylation is associated with suppression of inflammation [42,43,44]. Evaluation of the effect of coumarin derivative 2 on NF-κB p65 phosphorylation showed that it tended to inhibit p65 phosphorylation in a concentration-dependent manner (Figure 5). As a transcriptional regulator, the p65 protein induces an inflammatory response by regulating the expression of iNOS [49,50]. Coumarin derivative 2 decreased p65 phosphorylation, thereby inhibiting iNOS gene expression and reducing NO production. The inhibition of the NF-κB p65 signaling pathway is an important target for the treatment of inflammatory diseases and is considered to be an effective therapeutic strategy. Therefore, coumarin derivative 2 is predicted to exhibit anti-inflammatory effects by inhibiting p65 phosphorylation.

These results indicate the potential of coumarin derivative 2 in the prevention and treatment of immune diseases by targeting various inflammatory factors and diseases.

## 4. Materials and Methods

### 4.1. Antibodies and Reagents

Phosphorylation antibodies against ERK, JNK, p38, p65 and iNOS, glyceraldehyde 3-phosphate dehydrogenase, β-actin were obtained from Cell Signaling Technology (Danvers, MA, USA) (Table 3). TNF-α and mouse interleukin-6 (IL-6) ELISA kits were purchased from R&D Systems (Minneapolis, MN, USA). Lipopolysaccharides (LPS), phosphoric acid, sulfanilamide, and naphthyl ethylenediamine dihydrochloride were obtained from Sigma-Aldrich (St. Louis, MO, USA).

### 4.2. Cell Culture

Mouse macrophage RAW264.7 cells were purchased from the Korea Cell Line Bank (Seoul, Republic of Korea) and cultured in Dulbecco’s Modified Eagle Medium (Corning, NY, USA) containing 10% fetal bovine serum and 1% penicillin/streptomycin at 37 °C and 5% CO_2_. The medium was changed every 2 days and subcultured when the cells grew > 80%.

### 4.3. Cell Viability Assay

EZ-Cytox cell viability assay kit (Dogen, Suwon, Republic of Korea) was used to measure cell viability according to the manufacturer’s instructions. RAW264.7 cells were seeded into a 96-well plate (1 × 10^5^ cells/well) and treated with coumarin derivatives at concentrations of 20, 40, and 80 μM. After 2 h, the cells were treated with LPS (500 ng/mL) and cultured for 24 h in a 37 °C, 5% CO_2_ incubator. The culture supernatant was treated with 10% EZ-Cytox reagent, and a microplate reader was used to measure the absorbance at 450 nm.

### 4.4. Determination of NO

The Griess reagent was used to measure NO production. RAW264.7 cells were seeded into a 96-well plate and cultured for 24 h. The cells were treated with 20, 40, and 80 μM of coumarin derivatives, cultured for 2 h, and then cultured with LPS (500 ng/mL) for 20 h. The same amount of cell supernatant and Griess reagent were used for the reaction, and absorbance was measured at 540 nm.

### 4.5. Determination of TNF-α and IL-6

RAW264.7 cells were seeded into 96-well plates and cultured for 24 h. After 2 h of treatment with 20, 40, or 80 μM of coumarin compound derivative 2, the cells were incubated with LPS (500 ng/mL) for 20 h. ELISA kits (R & D Systems, Minneapolis, MN, USA) were used to collect and analyze the cell supernatants for TNF-α and IL-6.

### 4.6. Quantitative Real-Time Reverse Transcription Polymerase Chain Reaction (qRT-PCR)

RAW264.7 cells were seeded into a 6-well plate (2 × 10^6^ cells/well) and cultured for 24 h. The cells were treated with coumarin derivative 2 at 20, 40, and 80 μM and cultured for 2 h and with LPS (500 ng/mL) for 6 h.

The cells were washed with phosphate-buffered saline (PBS) and lysed with the RNeasy Mini kit (Qiagen, Hilden, Germany) for RNA isolation. RNA was converted to cDNA using the AccuPower reverse transcriptase Premix Kit (Bioneer, Daejeon, Republic of Korea). A Power SYBR Green PCR Master Mix (Applied Biosystems, Waltham, MN, USA) was used to perform polymerase chain reaction amplification. Amplification conditions were selected within the Quant 3 PCR system (Applied Biosystems). The data were normalized to the amount of glyceraldehyde 3-phosphate dehydrogenase.

### 4.7. Preparation of Cell Lysate and Immunoblotting

RAW264.7 cells were seeded into 6-well plates (2 × 10^6^ cells/well) and incubated for 24 h. The cells were incubated with 20, 40, and 80 μM of coumarin derivative 2 for 2 h, and then LPS (500 ng/mL) was added for 30 min (for phosphorylation of ERK, JNK, p38, and p65) and 24 h (for protein expression of iNOS).

The cells were washed with PBS and lysed with radioimmunoprecipitation assay buffer containing 1 mM dithiothreitol (Wako, Tokyo, Japan), phosphatase inhibitor cocktail, 1 mM phenylmethylsulfonylfluoride, 1 mM sodium orthovanadate (Sigma), and 10 mM β-glycerophosphate (Sigma). The extracted proteins were centrifuged at 13,000× *g* rpm, separated via sodium dodecyl sulfate polyacrylamide gel electrophoresis, and transferred to polyvinylidene fluoride membranes. Nonspecific proteins were blocked by adding 5% skim milk. After blocking, primary antibodies were incubated overnight at 4 °C. After washing with the PBS-T buffer, the secondary antibodies were treated at room temperature for 2 h. For detection, the antibodies were developed with Super Signal^®^ West Femto Substrate (ThermoFisher, Waltham, MN, USA) and analyzed on a Fusion Solo Chemiluminescence System (Vilber Lourmat, Paris, France) ECL detection system.

### 4.8. Statistical Analysis

The results of three independent experiments were expressed as the mean ± standard deviation (SD). One-way analysis of variance followed by Tukey’s post hoc test was used to perform all statistical analyses. The data are expressed as the mean ± SD. * *p* < 0.05 and ** *p* < 0.01 indicate statistical significance.

## Figures and Tables

**Table 1:** Cell viability of RAW264.7 cells treated with 23 coumarin derivatives.

	Control		LPS		Dexamethasone	
	Average (%)	SD	Average (%)	SD	Average (%)	SD
	100.0	0.7	97.4	1.9	102.5	1.8
Coumarin Derivative	20 μM		40 μM		80 μM	
	Average (%)	SD	Average (%)	SD	Average (%)	SD
1	90.43	2.65	96.02	2.22	109.35	2.98
2	106.05	7.64	97.13	0.00	100.77	14.46
3	95.37	5.31	100.88	1.08	110.15	5.85
4	79.89	5.74	91.19	2.55	88.66	0.92
5	86.79	1.52	109.69	0.32	113.21	0.65
6	98.66	14.52	110.92	8.34	110.61	2.82
7	105.59	1.68	121.03	1.52	124.32	7.37
8	103.10	5.96	106.55	0.32	93.49	3.20
9	118.45	0.85	111.03	0.91	112.10	1.09
10	110.82	0.00	113.30	3.28	104.16	2.12
11	113.13	2.55	108.24	4.01	122.49	3.88
12	99.18	0.79	101.85	1.64	105.92	1.21
13	113.78	6.25	117.64	1.03	112.23	3.82
14	109.53	10.80	95.75	1.64	101.67	3.10
15	108.58	1.46	111.67	4.98	113.69	1.88
16	106.95	3.88	101.80	0.49	96.27	3.46
17	122.42	6.34	121.99	3.55	136.60	0.07
18	129.27	2.39	129.70	0.27	137.99	3.27
19	117.31	2.39	119.48	2.45	119.72	0.20
20	114.22	6.34	123.92	5.86	135.00	11.73
21	130.57	0.68	122.08	1.09	102.27	1.02
22	120.20	3.48	121.65	0.34	113.60	1.50
23	119.14	0.34	117.94	0.55	116.68	0.68

Control: nontreated group; LPS: LPS (500 ng/mL) treated for 20 h; Dexamethasone: Dexamethasone (100 μM) treated for 2 h, and LPS treated for 20 h.

**Table 2:** NO production inhibitory effect of 23 coumarin derivatives in LPS-stimulated RAW264.7 cells.

	Control		LPS		Dexamethasone	
	Average (μM)	SD	Average (μM)	SD	Average (μM)	SD
	3.1	0.2	25.9	0.5	6.5 ^#^	0.8
	3.1	0.2	25.9	3.1	0.2	25.9
Coumarin Derivative	20 μM		40 μM		80 μM	
	Average (μM)	SD	Average (μM)	SD	Average (μM)	SD
1	29.50	1.34	29.82	0.45	28.71	2.02
2	22.53	1.79	15.24 ***	0.45	10.17 ***	0.45
3	27.60	1.34	28.86	1.34	22.84	0.45
4	15.71	0.22	17.45 ***	2.24	18.09 ***	2.24
5	30.77	3.14	28.71	0.67	25.38	0.45
6	30.92	0.67	26.49	0.67	16.82 ***	0.45
7	32.83	2.46	29.66	1.12	27.60	1.34
8	32.19	3.81	24.11	0.00	10.64 ***	0.67
9	23.27	0.59	24.10	2.17	21.32	0.98
10	24.24	0.00	20.34 **	0.00	9.21 ***	0.79
11	24.80	0.79	26.47	0.00	26.19	0.00
12	16.58	0.20	17.28 ***	0.79	17.98 ***	2.17
13	27.17	1.77	26.33	0.59	24.94	0.20
14	27.72	0.98	24.52	0.79	16.31 ***	0.20
15	26.33	0.59	22.71	1.38	19.37 **	0.59
16	27.58	1.97	26.33	0.59	16.17 ***	0.39
17	28.68	0.91	26.11	0.45	20.16 **	0.68
18	28.03	0.91	27.87	0.23	25.95	2.50
19	27.07	1.36	17.76	1.36	10.05 ***	0.45
20	31.89	1.82	30.12	0.23	19.52 **	1.59
21	28.68	1.82	22.73	2.95	11.97 ***	0.45
22	31.41	1.14	28.84	0.23	22.89	0.45
23	30.77	0.68	29.80	0.23	27.39	0.45

Control: nontreated group; LPS: LPS (500 ng/mL) treated for 20 h; Dexamethasone: Dexamethasone (100 μM) treated for 2 h and LPS treated for 20 h. ^#^ *p* < 0.0001 vs. the control group. *** p < 0.0001, and ** p < 0.001 vs. the LPS group.

**Table 3:** List of antibodies used in Western blotting analysis.

Antibodies	Source	Catalog Number
JNK	Cell Signaling Technology	#9252
ERK1/2	Cell Signaling Technology	#9102
p38	Cell Signaling Technology	#9212
p65	Cell Signaling Technology	#4764
p-JNK	Cell Signaling Technology	#9251
p-ERK1/2	Cell Signaling Technology	#9101
p-p38	Cell Signaling Technology	#4631
p-p65	Cell Signaling Technology	#3033
iNOS	Cell Signaling Technology	#13120
β-actin	Cell Signaling Technology	#4967
Anti-rabbit IgG	Cell Signaling Technology	#7074

## Data Availability

Not applicable.

## Supplementary Materials

The supporting information can be downloaded at: https://www.mdpi.com/article/10.3390/ijms241210026/s1.
