# Supplementary material for: Synthesis and Biological Properties of Pyranocoumarin Derivatives as Potent Anti-Inflammatory Agents
Source: Int J Mol Sci. 2023 Jun 12;24(12):10026. doi: 10.3390/ijms241210026 (PMC10298626; doi:10.3390/ijms241210026)
Supplement: Supplementary file 1 [file ijms-24-10026-s001.zip › ijms-2442620-supplementary.pdf]

Supporting information

Synthesis and Biological Properties of Pyranocoumarin Derivatives as Potent Anti-Inflammatory Agents

Su Ji Min [1], Heesu Lee [2], Myoung-Sook Shin [1*], and Jae Wook Lee [3*]

Experimental Section

General Information

Commercially available chemicals and solvents were used without further purification. Unless indicated otherwise, all reactions were conducted under an atmosphere of nitrogen. Removal of solvents and other volatile materials were concentrated under reduced pressure on rotary evaporator using a water bath temperature below 40 °C. Reaction was heated by IKA® RCT basic stir plate. Reaction was monitored by TLC (silica gel 60 F_{254}, Sigma Aldrich) and LC/MS (Agilent, LCMS-2020). Proton nuclear magnetic resonance (^1H NMR) spectra and Carbon13 nuclear magnetic resonance (^{13}C NMR) spectra were recorded at a 500MHz Superconducting LC-FT-NMR System : Varian. The proton signal for residual non-deuterated solvent (δ 2.50 for dimethyl sulfoxide) was used as an internal reference for ^1H NMR spectra. For ^{13}C NMR spectra, chemical shifts are reported relative to the δ 39.52 resonance of dimethyl sulfoxide. Coupling constants are reported in Hz. All compounds are >95% pure by HPLC analysis.

2-amino-4-(2-fluorophenyl)-5-oxo-4H,5H-pyrano[3,2-c]chromene-3-carbonitrile (1)

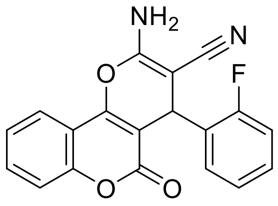

2-fluorobenzaldehyde (100 mg, 0.81 mmol) was dissolved in ethanol (5 mL). Then, malononitrile (53.5 mg, 0.81 mmol) and 4-hydroxycoumarin (131.3 mg, 0.81 mmol) was added sequentially and the reaction mixture was cooled in ice bath. And then, piperidine (10 µL) was added slowly and reaction mixture was warmed to room temperature slowly. The reaction mixture was stirred overnight. The reaction mixture was filtered, precipitate was collected. The precipitate was dried under air. **1** was collected as a white solid (268.3 mg, 80.3%). ^1H NMR (500 MHz, DMSO-d_6) δ 7.94 (dd, J = 7.95, 1.65 Hz, 1H), 7.71 (td, J = 8.7, 1.65 Hz, 1H), 7.49 (td, J = 8.75, 1 Hz, 1H), 7.46-7.42 (m, 3H), 7.30-7.26 (m, 2H), 4.71 (s, 1H). ^{13}C NMR (125 MHz, DMSO-d_6) δ 161.4, 159.8, 159.5, 158.5, 154.1, 152.3, 133.3, 130.4, 129.9, 129.5, 125.0, 122.7, 119.2, 116.8, 115.9, 113.0, 102.9, 56.6, 31.5. MS (m/z): $[M + H]^+$ calcd for $C_{19}H_{11}FN_2O_3$, 335.08; found 335.1.

2-amino-4-(3-fluorophenyl)-5-oxo-4H,5H-pyrano[3,2-c]chromene-3-carbonitrile (2)

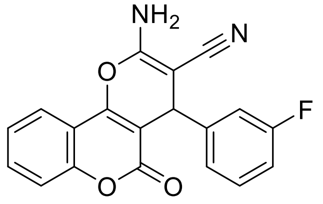

3-fluorobenzaldehyde (100 mg, 0.81 mmol) was dissolved in ethanol (5 mL). Then, malononitrile (53.5 mg, 0.81 mmol) and 4-hydroxycoumarin (131.3 mg, 0.81 mmol) was added sequentially and the reaction mixture was cooled in ice bath. And then, piperidine (10 μL) was added slowly and reaction mixture was warmed to room temperature slowly. The reaction mixture was stirred overnight. The reaction mixture was filtered, precipitate was collected. The precipitate was dried under air. **2** was collected as a white solid (251.4 mg, 76.1%). ^{1}H NMR (500 MHz, DMSO-d$_6$) δ 7.90 (d, *J* = 7.8 Hz, 1H), 7.72-7.68 (m, 1H), 7.49-7.46 (m, 1H), 7.46-7.43 (m, 3H), 7.35 (q, *J* = 7.55 Hz, 1H), 7.10-7.04 (m, 3H), 4.49 (s, 1H). ^{13}C NMR (125 MHz, DMSO-d$_6$) δ 163.4, 161.4, 159.9, 158.2, 154.0, 152.4, 146.4, 133.3, 130.7, 124.9, 124.0, 122.9, 119.3, 116.8, 114.8, 113.1, 103.5, 57.7, 36.8. MS (m/z): [M + H]$^+$ calcd for $C_{19}H_{11}FN_2O_3$, 335.08; found 335.1.

2-amino-4-(4-fluorophenyl)-5-oxo-4H,5H-pyrano[3,2-c]chromene-3-carbonitrile (3)

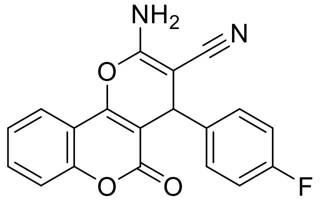

4-fluorobenzaldehyde (100 mg, 0.81 mmol) was dissolved in ethanol (5 mL). Then, malononitrile (53.5 mg, 0.81 mmol) and 4-hydroxycoumarin (131.3 mg, 0.81 mmol) was added sequentially and the reaction mixture was cooled in ice bath. And then, piperidine (10 μL) was added slowly and reaction mixture was warmed to room temperature slowly. The reaction mixture was stirred overnight. The reaction mixture was filtered, precipitate was collected. The precipitate was dried under air. **3** was collected as a white solid (232.5 mg, 70.0%). ^{1}H NMR (500 MHz, DMSO-d$_6$) δ 7.90 (d, *J* = 8.05 Hz, 1H), 7.75-7.68 (m, 1H), 7.50-7.43 (m, 2H), 7.43-7.40 (m, 2H), 7.30-7.28 (m, 2H), 7.12 (t, *J* = 8.6 Hz, 2H), 4.46 (s, 1H). ^{13}C NMR (125 MHz, DMSO-d$_6$) δ 162.4, 160.5, 159.8, 158.2, 153.7, 152.3, 139.7, 133.3, 129.9, 124.9, 122.8, 119.4, 116.8, 115.5, 115.3, 113.1, 104.0, 58.0, 36.4. MS (m/z): [M + H]$^+$ calcd for $C_{19}H_{11}FN_2O_3$, 335.08; found 335.1.

2-amino-5-oxo-4-(2-(trifluoromethyl)phenyl)-4H,5H-pyrano[3,2-c]chromene-3-carbonitrile (4)

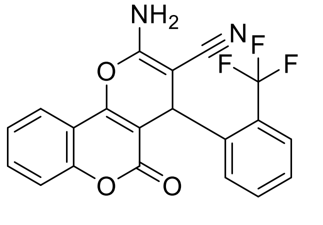

2-(trifluoromethyl)benzaldehyde (100 mg, 0.57 mmol) was dissolved in ethanol (5 mL). Then, malononitrile (37.7 mg, 0.57 mmol) and 4-hydroxycoumarin (92.4 mg, 0.57 mmol) was added sequentially and the reaction mixture was cooled in ice bath. And then, piperidine (10 μL) was added slowly and reaction mixture was warmed to room temperature slowly. The reaction mixture was stirred overnight. The reaction mixture was filtered, precipitate was collected. The precipitate was dried under air. **4** was collected as a white solid (152.4 mg, 70.2%). ^{1}H NMR (500 MHz, DMSO-d$_6$) δ 7.94 (dd, *J* = 7.95, 1.6 Hz, 1H), 7.73-7.68 (m, 2H), 7.58 (td, *J* = 7.6, 1.35 Hz,

1H), 7.49 (td, J = 7.55, 1.05 Hz, 1H), 7.45-7.41 (m, 5H), 4.83 (s, 1H). ^{13}C NMR (125 MHz, DMSO-d_6) δ 159.5, 158.2, 154.2, 152.4, 143.1, 133.3, 130.5, 127.8, 127.1, 126.9, 125.6, 124.9, 123.4, 122.9, 118.8, 116.7, 113.0, 103.8, 58.0, 32.9. MS (m/z): $[M + H]^+$ calcd for $C_{20}H_{11}F_3N_2O_3$, 385.07; found 385.1.

2-amino-5-oxo-4-(3-(trifluoromethyl)phenyl)-4H,5H-pyrano[3,2-c]chromene-3-carbonitrile (5)

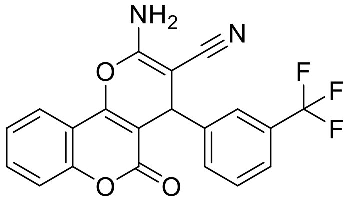

3-(trifluoromethyl)benzaldehyde (100 mg, 0.57 mmol) was dissolved in ethanol (5 mL). Then, malononitrile (37.7 mg, 0.57 mmol) and 4-hydroxycoumarin (92.4 mg, 0.57 mmol) was added sequentially and the reaction mixture was cooled in ice bath. And then, piperidine (10 μL) was added slowly and reaction mixture was warmed to room temperature slowly. The reaction mixture was stirred overnight. The reaction mixture was filtered, precipitate was collected. The precipitate was dried under air. **5** was collected as a white solid (181.5 mg, 82.9%). ^{1}H NMR (500 MHz, DMSO-d_6) δ 7.92 (d, J = 8.15 Hz, 1H), 7.73-7.70 (m, 1H), 7.62-7.57 (m, 2H), 7.57-7.53 (m, 2H), 7.50-7.44 (m, 4H), 4.63 (s, 1H). ^{13}C NMR (125 MHz, DMSO-d_6) δ 159.9, 158.3, 154.1, 152.4, 144.8, 133.3, 132.1, 130.0, 129.4, 129.1, 125.0, 124.2, 122.9, 119.2, 116.8, 113.1, 103.3, 57.5, 36.9. MS (m/z): $[M + H]^+$ calcd for $C_{20}H_{11}F_3N_2O_3$, 385.07; found 385.1.

2-amino-5-oxo-4-(4-(trifluoromethyl)phenyl)-4H,5H-pyrano[3,2-c]chromene-3-carbonitrile (6)

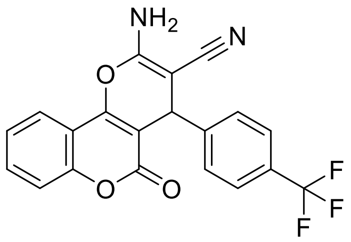

4-(trifluoromethyl)benzaldehyde (100 mg, 0.57 mmol) was dissolved in ethanol (5 mL). Then, malononitrile (37.7 mg, 0.57 mmol) and 4-hydroxycoumarin (92.4 mg, 0.57 mmol) was added sequentially and the reaction mixture was cooled in ice bath. And then, piperidine (10 μL) was added slowly and reaction mixture was warmed to room temperature slowly. The reaction mixture was stirred overnight. The reaction mixture was filtered, precipitate was collected. The precipitate was dried under air. **6** was collected as a white solid (179.5 mg, 82.0%). ^{1}H NMR (500 MHz, DMSO-d_6) δ 7.92 (dd, J = 7.9, 1.55 Hz, 1H), 7.72 (td, J = 8.7, 1.65 Hz, 1H), 7.67 (d, J = 8.1 Hz, 2H), 7.50-7.47 (m, 5H), 7.45 (d, J = 8.4 Hz, 1H), 4.58 (s, 1H). ^{13}C NMR (125 MHz, DMSO-d_6) δ 159.9, 158.2, 154.1, 152.4, 148.1, 133.4, 128.8, 128.1, 127.8, 125.7, 125.0, 122.9, 119.2, 116.8, 113.1, 103.3, 57.4, 37.0. MS (m/z): $[M + H]^+$ calcd for $C_{20}H_{11}F_3N_2O_3$, 385.07; found 385.1.

2-amino-4-(2-chlorophenyl)-5-oxo-4H,5H-pyrano[3,2-c]chromene-3-carbonitrile (7)

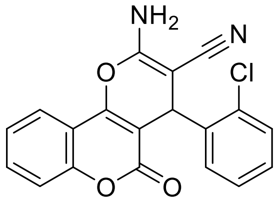

2-chlorobenzaldehyde (100 mg, 0.71 mmol) was dissolved in ethanol (5 mL). Then, malononitrile (46.9 mg, 0.71 mmol) and 4-hydroxycoumarin (115.1 mg, 0.71 mmol) was added sequentially and the reaction mixture was cooled in ice bath. And then, piperidine (10 μL) was added slowly and reaction mixture was warmed to room temperature slowly. The reaction mixture was stirred overnight. The reaction mixture was filtered, precipitate was collected. The precipitate was dried under air. **7** was collected as a white solid (164.4 mg, 66.0%). ^{1}H NMR (500 MHz, DMSO-d_6) δ 7.92 (dd, *J* = 7.9, 1.6 Hz, 1H), 7.71 (td, *J* = 8.8, 1.6 Hz, 1H), 7.49 (td, *J* = 7.6, 1.05 Hz, 1H), 7.45 (dd, *J* = 8.35, 0.95 Hz, 1H), 7.41-7.39 (m, 3H), 7.30-7.24 (m, 3H), 4.95 (s, 1H). ^{13}C NMR (125 MHz, DMSO-d_6) δ 159.7, 158.4, 154.3, 152.4, 140.3, 133.3, 132.6, 129.8, 129.1, 127.9, 125.0, 122.8, 119.0, 116.8, 113.0, 103.1, 56.7, 34.5. MS (m/z): $[M + H]^+$ calcd for $C_{19}H_{11}ClN_2O_3$, 351.05; found 351.1.

2-amino-4-(4-chlorophenyl)-5-oxo-4H,5H-pyrano[3,2-c]chromene-3-carbonitrile (8)

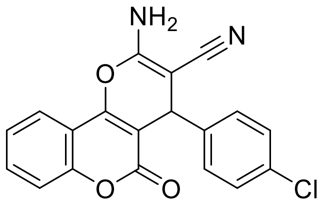

4-chlorobenzaldehyde (100 mg, 0.71 mmol) was dissolved in ethanol (5 mL). Then, malononitrile (46.9 mg, 0.71 mmol) and 4-hydroxycoumarin (115.1 mg, 0.71 mmol) was added sequentially and the reaction mixture was cooled in ice bath. And then, piperidine (10 μL) was added slowly and reaction mixture was warmed to room temperature slowly. The reaction mixture was stirred overnight. The reaction mixture was filtered, precipitate was collected. The precipitate was dried under air. **9** was collected as a white solid (203.1 mg, 81.6%). ^{1}H NMR (500 MHz, DMSO-d_6) δ 7.90 (dd, *J* = 7.95, 1.6 Hz, 1H), 7.70 (td, *J* = 7.4, 1.55 Hz, 1H), 7.48 (td, *J* = 7.6, 1.05 Hz, 1H), 7.45-7.43 (m, 3H), 7.37-7.34 (m, 2H), 7.29-7.27 (m, 2H), 4.47 (s, 1H). ^{13}C NMR (125 MHz, DMSO-d_6) δ 159.8, 158.2, 153.8, 152.4, 142.5, 133.3, 131.9, 129.8, 128.7, 125.0, 122.8, 119.3, 116.8, 113.1, 103.7, 57.7, 36.6. MS (m/z): $[M + H]^+$ calcd for $C_{19}H_{11}ClN_2O_3$, 351.05; found 351.1.

2-amino-5-oxo-4-(o-tolyl)-4H,5H-pyrano[3,2-c]chromene-3-carbonitrile (9)

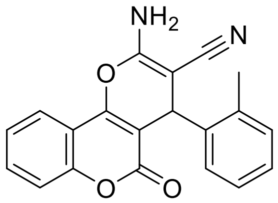

2-methylbenzaldehyde (100 mg, 0.83 mmol) was dissolved in ethanol (5 mL). Then, malononitrile (54.8 mg, 0.83 mmol) and 4-hydroxycoumarin (134.6 mg, 0.83 mmol) was added sequentially and the reaction mixture was cooled in ice bath. And then, piperidine (10 μL) was added slowly and reaction mixture was warmed to room temperature slowly. The reaction mixture was stirred overnight. The reaction mixture was filtered, precipitate was collected. The precipitate was dried under air. **10** was collected as a white solid (211.4 mg, 77.1%). ^{1}H NMR (500 MHz, DMSO-d_6) δ 7.92 (d, *J* = 7.95 Hz, 1H), 7.72-7.68 (m, 1H), 7.49 (t, *J* = 6.55 Hz, 1H), 7.44 (t, *J* = 8.55 Hz, 1H), 7.33 (s, 2H), 7.15-7.14 (m, 1H), 7.12-7.07 (m, 2H), 7.01 (d, *J* = 6.85 Hz, 1H), 4.75 (s, 1H), 2.47 (s, 3H). ^{13}C NMR (125 MHz, DMSO-d_6) δ 159.9, 158.0, 153.7, 152.2, 142.4, 135.5, 133.2, 130.3, 128.1, 127.0, 126.9, 125.0,

122.7, 119.5, 116.8, 113.1, 104.8, 58.1, 32.7, 19.2. MS (m/z): $[M + H]^+$ calcd for $C_{20}H_{14}N_2O_3$, 331.10; found 331.1.

2-amino-5-oxo-4-(m-tolyl)-4H,5H-pyrano[3,2-c]chromene-3-carbonitrile (10)

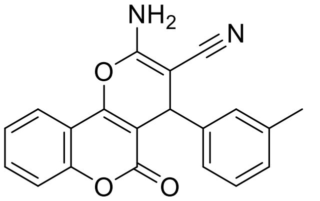

3-methylbenzaldehyde (100 mg, 0.83 mmol) was dissolved in ethanol (5 mL). Then, malononitrile (54.8 mg, 0.83 mmol) and 4-hydroxycoumarin (134.6 mg, 0.83 mmol) was added sequentially and the reaction mixture was cooled in ice bath. And then, piperidine (10 μL) was added slowly and reaction mixture was warmed to room temperature slowly. The reaction mixture was stirred overnight. The reaction mixture was filtered, precipitate was collected. The precipitate was dried under air. **11** was collected as a white solid (235.7 mg, 86.0%). ^{1}H NMR (500 MHz, DMSO-d_6) δ 7.91 (d, J = 7.9 Hz, 1H), 7.70 (q, J = 5.6 Hz, 1H), 7.49-7.42 (m, 2H), 7.36 (s, 2H), 7.19 (t, J = 7.75 Hz, 1H), 7.04-7.01 (m, 3H), 4.38 (s, 1H), 2.25 (s, 3H). ^{13}C NMR (500 MHz, DMSO-d_6) δ 159.8, 158.2, 153.6, 152.3, 143.5, 137.9, 133.2, 128.6, 128.2, 128.1, 125.0, 122.8, 119.5, 116.8, 113.1, 104.2, 58.3, 37.1, 21.2. MS (m/z): $[M + H]^+$ calcd for $C_{20}H_{14}N_2O_3$, 331.10; found 331.1.

2-amino-5-oxo-4-(p-tolyl)-4H,5H-pyrano[3,2-c]chromene-3-carbonitrile (11)

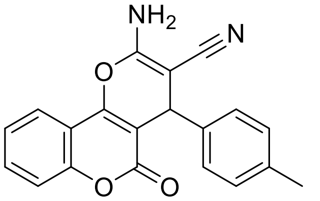

4-methylbenzaldehyde (100 mg, 0.83 mmol) was dissolved in ethanol (5 mL). Then, malononitrile (54.8 mg, 0.83 mmol) and 4-hydroxycoumarin (134.6 mg, 0.83 mmol) was added sequentially and the reaction mixture was cooled in ice bath. And then, piperidine (10 μL) was added slowly and reaction mixture was warmed to room temperature slowly. The reaction mixture was stirred overnight. The reaction mixture was filtered, precipitate was collected. The precipitate was dried under air. **12** was collected as a white solid (227.2 mg, 82.9%). ^{1}H NMR (500 MHz, DMSO-d_6) δ 7.90 (d, J = 7.8 Hz, 1H), 7.85-7.83 (m, 1H), 7.71-7.67 (m, 1H), 7.45-7.40 (m, 2H), 7.35 (s, 2H), 7.12-7.09 (m, 3H), 4.38 (s, 1H), 2.24 (s, 3H). ^{13}C NMR (500 MHz, DMSO-d_6) δ 159.8, 158.1, 153.5, 152.3, 146.0, 140.6, 136.6, 133.2, 130.9, 129.3, 127.7, 124.9, 122.7, 119.5, 116.7, 113.1, 104.3, 58.3, 36.7, 20.8. MS (m/z): $[M + H]^+$ calcd for $C_{20}H_{14}N_2O_3$, 331.10; found 331.1.

2-amino-5-oxo-4-(2-(trifluoromethoxy)phenyl)-4H,5H-pyrano[3,2-c]chromene-3-carbonitrile (12)

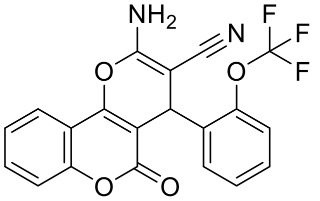

2-(trifluoromethoxy)benzaldehyde (100 mg, 0.53 mmol) was dissolved in ethanol (5 mL). Then, malononitrile

(35.0 mg, 0.53 mmol) and 4-hydroxycoumarin (86.0 mg, 0.53 mmol) was added sequentially and the reaction mixture was cooled in ice bath. And then, piperidine (10 μL) was added slowly and reaction mixture was warmed to room temperature slowly. The reaction mixture was stirred overnight. The reaction mixture was filtered, precipitate was collected. The precipitate was dried under air. **13** was collected as a white solid (198.4 mg, 93.5%). ^{1}H NMR (500 MHz, DMSO-d$_6$) δ 7.91 (dd, *J* = 7.95, 1.6 Hz, 1H), 7.70 (td, *J* = 7.45, 1.65 Hz, 1H), 7.47 (t, *J* = 7.45 Hz, 1H), 7.46-7.37 (m, 5H), 7.32-7.28 (m, 2H), 4.73 (s, 1H). ^{13}C NMR (125 MHz, DMSO-d$_6$) δ 159.7, 158.5, 154.1, 152.3, 147.2, 134.4, 133.3, 131.5, 129.5, 127.4, 125.0, 122.7, 119.5, 119.1, 116.8, 113.0, 102.9, 56.4, 33.1. MS (m/z): [M + H]$^+$ calcd for $C_{20}H_{11}F_3N_2O_4$, 401.07; found 401.1.

2-amino-5-oxo-4-(3-(trifluoromethoxy)phenyl)-4H,5H-pyrano[3,2-c]chromene-3-carbonitrile (13)

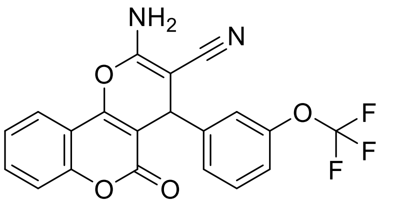

3-(trifluoromethoxy)benzaldehyde (100 mg, 0.53 mmol) was dissolved in ethanol (5 mL). Then, malononitrile (35.0 mg, 0.53 mmol) and 4-hydroxycoumarin (86.0 mg, 0.53 mmol) was added sequentially and the reaction mixture was cooled in ice bath. And then, piperidine (10 μL) was added slowly and reaction mixture was warmed to room temperature slowly. The reaction mixture was stirred overnight. The reaction mixture was filtered, precipitate was collected. The precipitate was dried under air. **14** was collected as a white solid (181.5 mg, 85.5%). ^{1}H NMR (500 MHz, DMSO-d$_6$) δ 7.90 (dd, *J* = 7.95, 1.6 Hz, 1H), 7.70 (td, *J* = 7.4, 1.6 Hz, 1H), 7.48-7.42 (m, 5H), 7.29 (d, *J* = 7.8 Hz, 1H), 7.25 (s, 1H), 7.22 (d, *J* = 8.2 Hz, 1H), 4.55 (s, 1H). ^{13}C NMR (125 MHz, DMSO-d$_6$) δ 159.9, 158.3, 154.1, 152.4, 148.6, 146.2, 133.3, 130.7, 126.9, 124.9, 122.9, 120.5, 119.7, 119.2, 116.8, 113.1, 103.3, 57.5, 36.8. MS (m/z): [M + H]$^+$ calcd for $C_{20}H_{11}F_3N_2O_4$, 401.07; found 401.1.

2-amino-5-oxo-4-(4-(trifluoromethoxy)phenyl)-4H,5H-pyrano[3,2-c]chromene-3-carbonitrile (14)

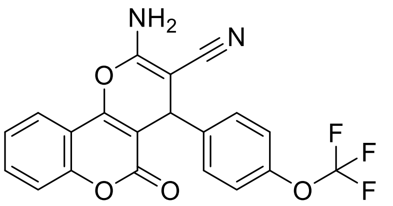

4-(trifluoromethoxy)benzaldehyde (100 mg, 0.53 mmol) was dissolved in ethanol (5 mL). Then, malononitrile (35.0 mg, 0.53 mmol) and 4-hydroxycoumarin (86.0 mg, 0.53 mmol) was added sequentially and the reaction mixture was cooled in ice bath. And then, piperidine (10 μL) was added slowly and reaction mixture was warmed to room temperature slowly. The reaction mixture was stirred overnight. The reaction mixture was filtered, precipitate was collected. The precipitate was dried under air. **15** was collected as a white solid (201.4 mg, 94.9%). ^{1}H NMR (500 MHz, DMSO-d$_6$) δ 7.91 (dd, *J* = 7.95, 1.6 Hz, 1H), 7.71 (td, *J* = 7.45, 1.65 Hz, 1H), 7.48 (t, *J* = 7.6 Hz, 1H), 7.44 (t, *J* = 3.95 Hz, 3H), 7.39 (d, *J* = 8.65 Hz, 2H), 7.29 (d, *J* = 8.25 Hz, 2H), 4.52 (s, 1H). ^{13}C NMR (125 MHz, DMSO-d$_6$) δ 159.9, 158.2, 153.9, 152.4, 147.5, 142.9, 133.3, 129.8, 125.0, 122.8, 121.3, 119.3, 116.8, 113.1, 103.7, 57.7, 36.6. MS (m/z): [M + H]$^+$ calcd for $C_{20}H_{11}F_3N_2O_4$, 401.07; found 401.1.

2-amino-4-(2-methoxyphenyl)-5-oxo-4H,5H-pyrano[3,2-c]chromene-3-carbonitrile (15)

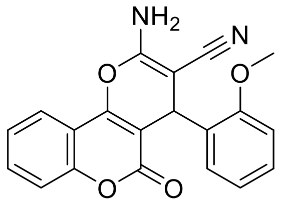

2-methoxybenzaldehyde (100 mg, 0.74 mmol) was dissolved in ethanol (5 mL). Then, malononitrile (48.9 mg, 0.74 mmol) and 4-hydroxycoumarin (120.0 mg, 0.74 mmol) was added sequentially and the reaction mixture was cooled in ice bath. And then, piperidine (10 µL) was added slowly and reaction mixture was warmed to room temperature slowly. The reaction mixture was stirred overnight. The reaction mixture was filtered, precipitate was collected. The precipitate was dried under air. **16** was collected as a white solid (190.5 mg, 74.3%). ^{1}H NMR (500 MHz, DMSO-d$_6$) δ 7.92 (dd, *J* = 8.15, 1.6 Hz, 1H), 7.68 (td, *J* = 7.35, 1.65 Hz, 1H), 7.47 (t, *J* = 7.7 Hz, 1H), 7.43 (d, *J* = 8.35 Hz, 1H), 7.23-7.19 (m, 3H), 7.08 (dd, *J* = 7.6, 1.75 Hz, 1H), 6.97 (d, *J* = 7.25 Hz, 1H), 6.86 (t, *J* = 7.45 Hz, 1H), 4.69 (s, 1H), 3.69 (s, 3H). ^{13}C NMR (125 MHz, DMSO-d$_6$) δ 159.8, 158.8, 157.4, 154.3, 152.3, 133.0, 130.9, 129.3, 128.8, 124.9, 122.6, 120.8, 119.6, 116.7, 113.2, 112.0, 103.5, 57.1, 56.0, 32.4. MS (m/z): [M + H]$^+$ calcd for $C_{20}H_{14}N_2O_4$, 347.10; found 347.1.

2-amino-4-(3-methoxyphenyl)-5-oxo-4H,5H-pyrano[3,2-c]chromene-3-carbonitrile (16)

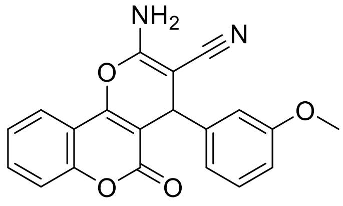

3-methoxybenzaldehyde (100 mg, 0.74 mmol) was dissolved in ethanol (5 mL). Then, malononitrile (48.9 mg, 0.74 mmol) and 4-hydroxycoumarin (120.0 mg, 0.74 mmol) was added sequentially and the reaction mixture was cooled in ice bath. And then, piperidine (10 µL) was added slowly and reaction mixture was warmed to room temperature slowly. The reaction mixture was stirred overnight. The reaction mixture was filtered, precipitate was collected. The precipitate was dried under air. **17** was collected as a white solid (204.7 mg, 79.9%). ^{1}H NMR (500 MHz, DMSO-d$_6$) δ 7.90 (dd, *J* = 7.95, 1.6 Hz, 1H), 7.69 (td, *J* = 7.35, 1.6 Hz, 1H), 7.46 (t, *J* = 7.6 Hz, 1H), 7.42 (d, *J* = 8.35 Hz, 1H), 7.38 (s, 2H), 7.22 (t, *J* = 7.85 Hz, 1H), 6.82-6.77 (m, 3H), 4.41 (s, 1H), 3.70 (s, 3H). ^{13}C NMR (125 MHz, DMSO-d$_6$) δ 159.8, 159.5, 158.2, 153.7, 152.3, 145.1, 133.2, 130.0, 124.9, 122.8, 119.9, 119.4, 116.8, 114.1, 113.1, 112.2, 104.0, 58.1, 55.2, 37.1. MS (m/z): [M + H]$^+$ calcd for $C_{20}H_{14}N_2O_4$, 347.10; found 347.1.

2-amino-4-(4-methoxyphenyl)-5-oxo-4H,5H-pyrano[3,2-c]chromene-3-carbonitrile (17)

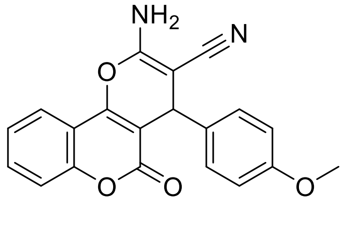

4-methoxybenzaldehyde (100 mg, 0.74 mmol) was dissolved in ethanol (5 mL). Then, malononitrile (48.9 mg, 0.74 mmol) and 4-hydroxycoumarin (120.0 mg, 0.74 mmol) was added sequentially and the reaction mixture was cooled in ice bath. And then, piperidine (10 µL) was added slowly and reaction mixture was warmed to room temperature slowly. The reaction mixture was stirred overnight. The reaction mixture was filtered, precipitate was collected. The precipitate was dried under air. **18** was collected as a white solid (202.3 mg, 78.9%). ^{1}H NMR (500 MHz, DMSO-d$_6$) δ 7.92 (dd, *J* = 7.95, 1.55 Hz, 1H), 7.72 (td, *J* = 7.4, 1.6 Hz, 1H), 7.67 (d, *J* = 8.2 Hz, 2H), 7.50-7.44 (m, 6H), 4.58 (s, 1H), 3.50 (s, 3H). ^{13}C NMR (125 MHz, DMSO-d$_6$) δ 159.9, 158.2, 154.1, 152.4, 148.1,

133.4, 128.8, 128.1, 127.8, 125.7, 125.0, 122.9, 119.2, 116.8, 113.1, 103.3, 57.4, 37.0. MS (m/z): $[M + H]^+$ calcd for $C_{20}H_{14}N_2O_4$, 347.10; found 347.1.

2-amino-4-(2-bromophenyl)-5-oxo-4H,5H-pyrano[3,2-c]chromene-3-carbonitrile (18)

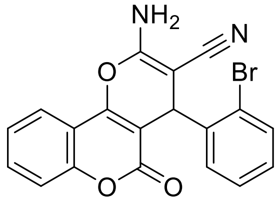

2-bromobenzaldehyde (100 mg, 0.54 mmol) was dissolved in ethanol (5 mL). Then, malononitrile (35.7 mg, 0.54 mmol) and 4-hydroxycoumarin (87.6 mg, 0.54 mmol) was added sequentially and the reaction mixture was cooled in ice bath. And then, piperidine (10 μL) was added slowly and reaction mixture was warmed to room temperature slowly. The reaction mixture was stirred overnight. The reaction mixture was filtered, precipitate was collected. The precipitate was dried under air. **19** was collected as a white solid (205.8 mg, 96.4%). ^{1}H NMR (500 MHz, DMSO-d$_6$) δ 7.91 (dd, *J* = 7.9, 1.6 Hz, 1H), 7.71 (td, *J* = 7.45, 1.65 Hz, 1H), 7.57 (d, *J* = 8 Hz, 1H), 7.49 (t, *J* = 7.6 Hz, 1H), 7.41 (s, 2H), 7.31 (t, *J* = 7.6 Hz, 1H), 7.26 (d, *J* = 7.8 Hz, 1H), 7.17 (td, *J* = 7.95, 1.85 Hz, 1H), 4.98 (s, 1H). ^{13}C NMR (125 MHz, DMSO-d$_6$) δ 159.7, 158.3, 154.3, 152.4, 133.3, 133.0, 129.3, 128.6, 125.0, 123.1, 122.8, 118.9, 116.8, 113.0, 103.3, 56.9, 36.7. MS (m/z): $[M + H]^+$ calcd for $C_{19}H_{11}BrN_2O_3$, 395.00; found 395.0.

2-amino-4-(3-bromophenyl)-5-oxo-4H,5H-pyrano[3,2-c]chromene-3-carbonitrile (19)

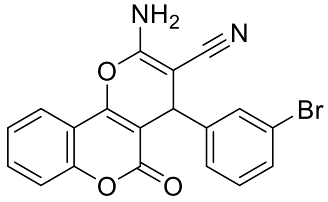

3-bromobenzaldehyde (100 mg, 0.54 mmol) was dissolved in ethanol (5 mL). Then, malononitrile (35.7 mg, 0.54 mmol) and 4-hydroxycoumarin (87.6 mg, 0.54 mmol) was added sequentially and the reaction mixture was cooled in ice bath. And then, piperidine (10 μL) was added slowly and reaction mixture was warmed to room temperature slowly. The reaction mixture was stirred overnight. The reaction mixture was filtered, precipitate was collected. The precipitate was dried under air. **20** was collected as a white solid (193.4 mg, 90.6%). ^{1}H NMR (500 MHz, DMSO-d$_6$) δ 7.90 (dd, *J* = 7.95, 1.55 Hz, 1H), 7.71 (td, *J* = 7.5, 1.65 Hz, 1H), 7.48 (t, *J* = 7.65 Hz, 1H), 7.45-7.42 (m, 5H), 7.30-7.27 (m, 2H), 4.48 (s, 1H). ^{13}C NMR (125 MHz, DMSO-d$_6$) δ 159.9, 158.2, 154.0, 152.4, 146.2, 133.3, 131.0, 130.6, 130.3, 127.1, 125.0, 122.9, 121.9, 119.3, 116.8, 113.1, 103.4, 57.6, 36.8. MS (m/z): $[M + H]^+$ calcd for $C_{19}H_{11}BrN_2O_3$, 395.00; found 395.0.

2-amino-4-(4-bromophenyl)-5-oxo-4H,5H-pyrano[3,2-c]chromene-3-carbonitrile (20)

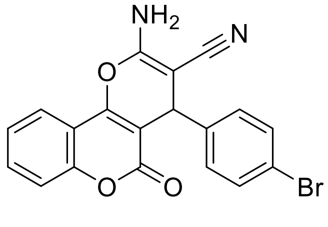

4-bromobenzaldehyde (100 mg, 0.54 mmol) was dissolved in ethanol (5 mL). Then, malononitrile (35.7 mg, 0.54 mmol) and 4-hydroxycoumarin (87.6 mg, 0.54 mmol) was added sequentially and the reaction mixture was cooled in ice bath. And then, piperidine (10 μL) was added slowly and reaction mixture was warmed to room temperature slowly. The reaction mixture was stirred overnight. The reaction mixture was filtered, precipitate was collected. The precipitate was dried under air. **21** was collected as a white solid (182.5 mg, 85.5%). ^{1}H NMR (500 MHz, DMSO-d_6) δ 7.90 (dd, *J* = 7.9, 1.55 Hz, 1H), 7.85-7.81 (m, 3H), 7.71-7.68 (m, 1H), 7.49-7.47 (m, 1H), 7.47-7.43 (m, 1H), 7.22 (d, *J* = 8.45 Hz, 1H), 4.45 (s, 1H). ^{13}C NMR (125 MHz, DMSO-d_6) δ 160.5, 159.8, 158.2, 153.8, 152.4, 142.9, 133.3, 132.9, 132.3, 131.6, 130.5, 130.2, 128.5, 125.0, 122.8, 120.5, 116.8, 114.3, 113.2, 113.1, 82.6, 57.7, 44.0, 36.7. MS (m/z): $[M + H]^+$ calcd for $C_{19}H_{11}BrN_2O_3$, 395.00; found 395.0.

2-amino-4-(3-cyanophenyl)-5-oxo-4H,5H-pyrano[3,2-c]chromene-3-carbonitrile (21)

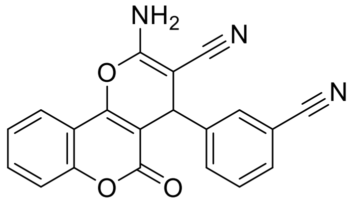

3-cyanobenzaldehyde (100 mg, 0.76 mmol) was dissolved in ethanol (5 mL). Then, malononitrile (50.2 mg, 0.76 mmol) and 4-hydroxycoumarin (123.2 mg, 0.76 mmol) was added sequentially and the reaction mixture was cooled in ice bath. And then, piperidine (10 μL) was added slowly and reaction mixture was warmed to room temperature slowly. The reaction mixture was stirred overnight. The reaction mixture was filtered, precipitate was collected. The precipitate was dried under air. **22** was collected as a white solid (176.8 mg, 68.1%). ^{1}H NMR (500 MHz, DMSO-d_6) δ 7.91 (dd, *J* = 7.95, 1.6 Hz, 1H), 7.79 (s, 1H), 7.71-7.68 (m, 2H), 7.64 (dt, *J* = 8.2, 1.4 Hz, 1H), 7.53 (t, *J* = 7.75 Hz, 1H), 7.48-7.45 (m, 3H), 7.43 (d, *J* = 8.4 Hz, 1H), 4.57 (s, 1H). ^{13}C NMR (125 MHz, DMSO-d_6) δ 159.9, 158.2, 154.3, 152.5, 145.1, 133.3, 133.1, 131.7, 131.3, 129.9, 124.9, 122.9, 119.2, 118.9, 116.7, 113.2, 111.6, 102.9, 57.4, 36.8. MS (m/z): $[M + H]^+$ calcd for $C_{20}H_{11}N_3O_3$, 342.08; found 342.1.

2-amino-4-(4-cyanophenyl)-5-oxo-4H,5H-pyrano[3,2-c]chromene-3-carbonitrile (22)

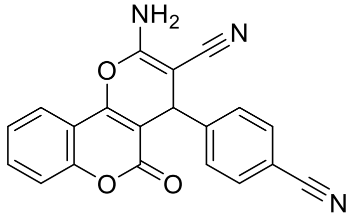

4-cyanobenzaldehyde (100 mg, 0.76 mmol) was dissolved in ethanol (5 mL). Then, malononitrile (50.2 mg, 0.76 mmol) and 4-hydroxycoumarin (123.2 mg, 0.76 mmol) was added sequentially and the reaction mixture was cooled in ice bath. And then, piperidine (10 μL) was added slowly and reaction mixture was warmed to room temperature slowly. The reaction mixture was stirred overnight. The reaction mixture was filtered, precipitate was collected. The precipitate was dried under air. **23** was collected as a white solid (191.2 mg, 73.7%). ^{1}H NMR (500 MHz, DMSO-d_6) δ 7.91 (dd, *J* = 7.9, 1.65 Hz, 1H), 7.78 (d, *J* = 8.35 Hz, 2H), 7.72 (td, *J* = 7.45, 1.65 Hz, 1H), 7.50-7.47 (m, 5H), 7.45 (d, *J* = 8.4 Hz, 1H), 4.58 (s, 1H). ^{13}C NMR (125 MHz, DMSO-d_6) δ 159.9, 158.3, 154.2, 152.4, 149.0, 133.4, 132.7, 129.1, 125.0, 122.9, 119.2, 118.9, 116.8, 113.1, 110.2, 103.0, 58.1, 37.0. MS (m/z): $[M + H]^+$ calcd for $C_{20}H_{11}N_3O_3$, 342.08; found 342.1.

2-amino-5-oxo-4-phenyl-4H,5H-pyrano[3,2-c]chromene-3-carbonitrile (23)

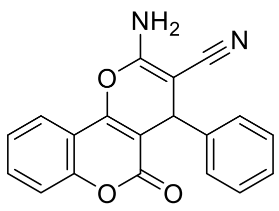

Benzaldehyde (100 mg, 0.94 mmol) was dissolved in ethanol (5 mL). Then, malononitrile (62.1 mg, 0.94 mmol) and 4-hydroxycoumarin (152.4 mg, 0.94 mmol) was added sequentially and the reaction mixture was cooled in ice bath. And then, piperidine (10 μL) was added slowly and reaction mixture was warmed to room temperature slowly. The reaction mixture was stirred overnight. The reaction mixture was filtered, precipitate was collected. The precipitate was dried under air. **24** was collected as a white solid (211.5 mg, 71.1%). ^{1}H NMR (500 MHz, DMSO-d_6) δ 7.91 (dd, J = 7.95, 1.6 Hz, 1H), 7.70 (td, J = 7.35, 1.6 Hz, 1H), 7.47 (t, J = 7.6 Hz, 1H), 7.44 (d, J = 8.35 Hz, 1H), 7.38 (s, 2H), 7.32-7.29 (m, 2H), 7.24-7.23 (m, 3H), 4.43 (s, 1H). ^{13}C NMR (125 MHz, DMSO-d_6) δ 159.9, 158.2, 153.7, 152.3, 143.5, 133.3, 128.8, 127.8, 127.4, 125.0, 122.8, 119.5, 116.8, 113.1, 104.2, 58.2, 37.2. MS (m/z): $[M + H]^+$ calcd for $C_{19}H_{12}N_2O_3$, 342.08; found 342.1.
